# Follicle-stimulating hormone promotes EndMT in endothelial cells by upregulating ALKBH5 expression

**DOI:** 10.1186/s11658-025-00720-y

**Published:** 2025-04-04

**Authors:** Ping Li, Yixiao Xiang, Jinzhi Wei, Xingyan Xu, Jiale Wang, Haowei Yu, Xiaosa Li, Huiping Lin, Xiaodong Fu

**Affiliations:** 1 The Affiliated Qingyuan Hospital (Qingyuan People’s Hospital), Guangzhou Medical University, Qingyuan, 511518 Guangdong People’s Republic of China; 2 Key Laboratory of Cardiovascular Diseases, Guangzhou Municipal and Guangdong Provincial Key Laboratory of Protein Modification and Degradation, School of Basic Medical Sciences, Guangzhou Medical University, Guangzhou, 511436 Guangdong People’s Republic of China; 3 Department of Cardiology, Guangzhou Institute of Cardiovascular Disease, Guangdong Key Laboratory of Vascular Diseases, The Second Affiliated Hospital, Guangzhou Medical University, Guangzhou, 510260 Guangdong People’s Republic of China

**Keywords:** Follicle-stimulating hormone (FSH), Endothelial to mesenchymal transition (EndMT), FOXM1, ALKBH5

## Abstract

**Background:**

The incidence of atherosclerosis markedly rises following menopause. Our previous findings demonstrated that elevated follicle-stimulating hormone (FSH) levels in postmenopausal women accelerate atherosclerosis progression. Plaque instability, the fundamental pathological factor in acute coronary syndrome, primarily results from vascular embolism due to plaque rupture. Recent evidence highlights that endothelial-to-mesenchymal transition (EndMT) exacerbates plaque instability, although the link between FSH and EndMT has not been fully established. This investigation sought to explore the possible influence of FSH in modulating EndMT.

**Methods:**

In this study, *apolipoprotein E*-deficient (*ApoE*^−/−^) mice served as an atherosclerosis model, while human umbilical vascular endothelial cells (HUVECs) were used as cellular models. Protein levels were assessed through immunochemical techniques, gene expression was quantified via RT-qPCR, and nucleic acid–protein interactions were evaluated using immunoprecipitation. The m6A modification status was determined by MeRIP, and cellular behaviors were analyzed through standard biochemical assays.

**Results:**

Our results indicate that FSH induces EndMT both in vitro and in vivo. Additional investigation suggested that FSH upregulates the transcription factor Forkhead box protein M1 (FOXM1) at both protein and mRNA levels by enhancing the expression of AlkB homolog 5, RNA demethylase (ALKBH5). FSH reduces m6A modifications on *FOXM1* through ALKBH5, leading to increased nascent transcript levels and mRNA stability of *FOXM1*. Dual-luciferase reporter assays highlighted cAMP-response element binding protein (CREB)’s essential function in facilitating the FSH-induced upregulation of ALKBH5.

**Conclusions:**

These findings suggest that FSH promotes ALKBH5 expression, facilitates *N*^6^-methyladenosine (m6A) demethylation on *FOXM1*, and consequently, induces EndMT. This study elucidates the impact of FSH on plaque instability and provides insights into potential strategies to prevent acute coronary syndrome in postmenopausal women.

**Graphical Abstract:**

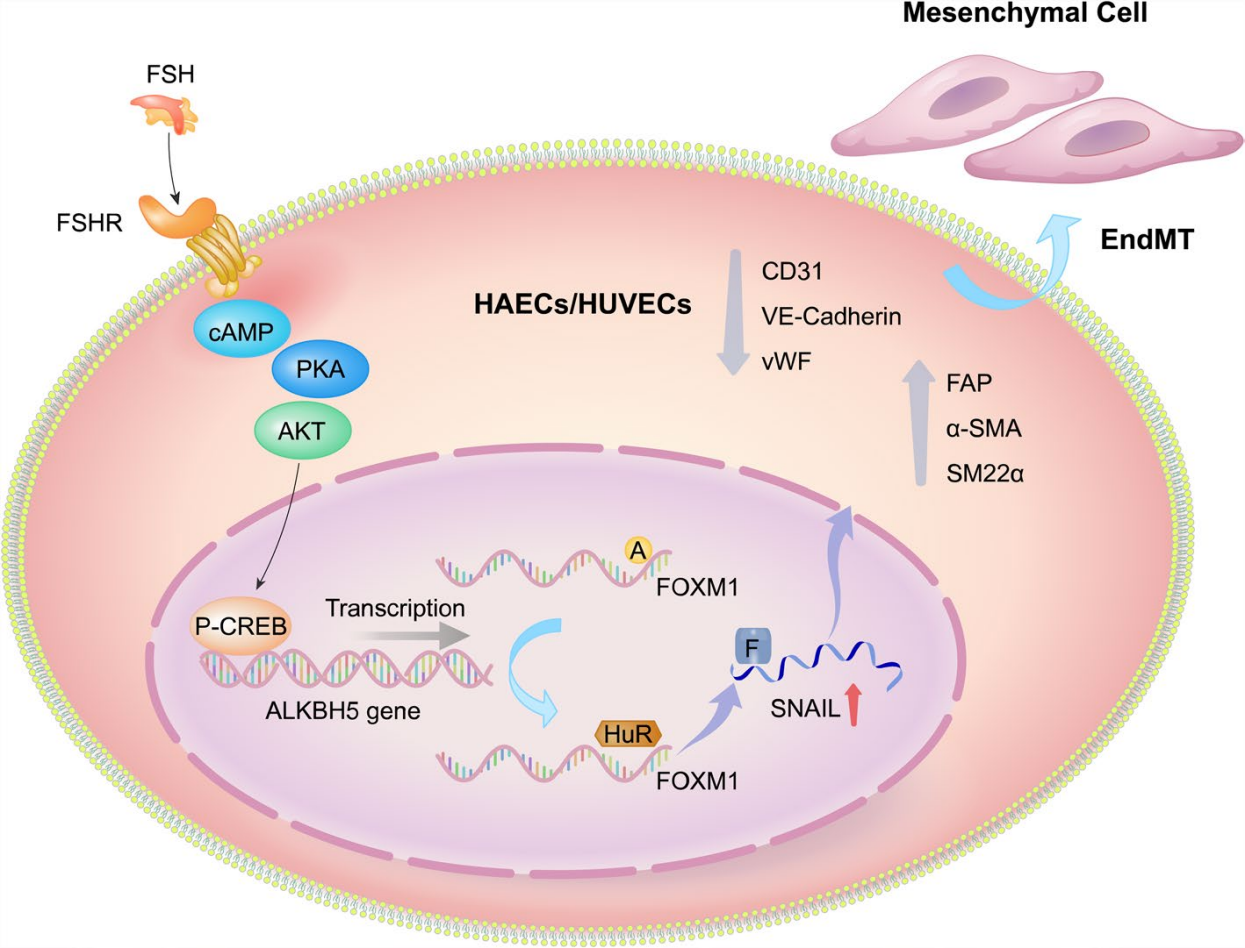

**Supplementary Information:**

The online version contains supplementary material available at 10.1186/s11658-025-00720-y.

## Background

Atherosclerotic cardiovascular disease represents the primary factor behind angina, myocardial infarction, stroke, and peripheral artery disease, collectively accounting for a significant proportion of global mortality [1]. The incidence of atherosclerosis is markedly lower in premenopausal women compared with age-matched men; however, this disparity diminishes after menopause, with women’s cardiovascular disease (CVD) rates rising markedly and eventually equaling those of men, underscoring the critical influence of sex hormones on atherosclerosis incidence [2]. Estrogen (E2) is well-known for its cardioprotective effects, both directly and indirectly, on the vasculature. Several clinical studies have suggested that estrogen replacement therapy (ERT) reduces the incidence of atherosclerotic diseases in postmenopausal women, although the overall efficacy of ERT remains debated [2, 3]. Several studies indicate that ERT does not markedly reduce cardiovascular mortality postmenopause, particularly in women over 60 years of age, and it carries the risk of inducing breast and endometrial cancers [4, 5]. Thus, identifying new risk factors in postmenopausal women is essential to developing novel therapeutic targets for effective prevention.

During menopause, the most prominent hormonal changes include a decrease in estrogen (E2, from 60.26 pg/mL to 19.12 pg/mL) and an increase in follicle-stimulating hormone (FSH, from 15.15 mIU/mL to 98.21 mIU/mL) [6]. Despite limited attention to FSH’s effects on the cardiovascular system, emerging clinical evidence, such as findings from the Study of Women’s Health Across the Nation (SWAN), indicates that elevated postmenopausal FSH levels markedly increase carotid artery intima thickness [7]. Furthermore, women with lower FSH elevations during menopause have a reduced risk of atherosclerosis compared with those with higher FSH levels [8]. These findings align with our previous research, which demonstrated that FSH accelerates atherogenesis by upregulating endothelial VCAM-1 expression, suggesting that targeting FSH could offer a novel approach to preventing CVD in postmenopausal women [9]. Indeed, the potential of FSH intervention is supported by evidence showing that FSH antibody administration effectively prevents osteoporosis in postmenopausal women [10, 11].

Endothelial-to-mesenchymal transition (EndMT) is prevalent in atherosclerotic plaques and is strongly associated with plaque instability [12]. Sharing similarities with EndMT, epithelial-to-mesenchymal transition (EMT) can be induced by FSH in cancer cells [13], though the underlying mechanisms require further investigation. Interestingly, recent studies suggest that mRNA *N*^6^-methyladenosine (m6A) modification serves a crucial function in EMT across various cancers, and m6A modification is also pivotal in atherosclerosis development [14]. For instance, m6A demethyltransferase alkB homolog 5, RNA demethylase (ALKBH5) inhibits tumor necrosis factor alpha (TNF-α)-induced apoptosis of human umbilical vascular endothelial cells (HUVECs) through the Bcl-2 pathway [15], and it helps maintain endothelial cell angiogenesis after acute ischemic stress by reducing *SPHK1* m6A methylation and downstream eNOS-Akt [16]. Evidently, ALKBH5 can regulate various functions of vascular endothelial cells, thereby influencing the initiation and progression of atherosclerosis. In addition, the transcription factor *Forkhead box protein M1* (*FOXM1*), as a target gene of ALKBH5 [17, 18] can regulate EMT in various tumors [19–21] and EndMT in endothelial cells [22, 23]. Thus, exploring whether FSH influences EndMT via ALKBH5/FOXM1 presents an intriguing and clinically significant scientific inquiry.

This study investigated FSH’s role in promoting EndMT both in vitro and within atherosclerotic artery walls in vivo and explored the contribution of m6A modification to these effects. Our findings reveal that FSH facilitates EndMT and atherosclerotic plaque formation in *ApoE*^−/−^ mice. Mechanistically, FSH upregulates the demethylase ALKBH5, reducing m6A levels on the transcription factor *FOXM1* mRNA, which in turn enhances FOXM1 protein expression and promotes EndMT. These insights offer a potential new approach for preventing and delaying atherosclerosis progression and clinical acute cardiovascular events in postmenopausal women.

## Materials and methods

### Reagents and antibodies

FSH (no. F4021), Oil Red O (ORO) (no. O0625), phenylmethanesulfonyl fluoride (PMSF) (no. 78830), protease inhibitor cocktail (PIC) (no. P8340), phosphatase inhibitor cocktail (PHIC) (no. P0044), NaF (no. S6776), and penicillin–streptomycin solution (no. V900929) were acquired from Sigma-Aldrich (St. Louis, MO, USA). Cluster of differentiation (CD)31 (no. 2528S), von Willebrand factor (vWF) (no. 65707S), vascular endothelial (VE)-cadherin (no. 2500S), alpha smooth muscle actin (α-SMA) (no. 19245S), fibroblast activation protein (FAP) (no. 66562S), FOXM1 (no. 20459S), SNAIL (no. 3879S), SLUG (no. 9585S), and Twist1 (no. 90445S), total-CREB (no. 9104S), and phospho-CREB (Ser133) (no. 9198S) antibodies were obtained from Cell Signaling Technology (Danvers, MA, USA). SM22α (no. ab14106) and ALKBH5 (no. ab195377) were purchased from Abcam (Boston, MA, USA); GAPDH (no. SC-47724) was acquired from Santa Cruz Biotechnology (Dallas, TX, USA). Phospho-CREB (Ser129, Ser133) (no. 44297G) antibody was procured from Thermo Fisher Scientific (Rockford, IL, USA). Actinomycin D (no. HY-17559) was sourced from MedChemExpress (NJ, USA). The Magna MeRIP™ m6A Kit (no. 17-371) and RNA immunoprecipitation (RIP) Kit (no. 17-700) were purchased from Millipore (MA, USA). Luciferase reporter gene test kit (no. E1910) was obtained from Promega (Beijing, China).

### Animals

The 8-to-10-week-old female *ApoE*-deficient (*ApoE*^−/−^) mice utilized in this investigation were acquired from Beijing Vital River Laboratory Animal Technology Co., Ltd. (Beijing, China). The study protocols for animal research were sanctioned by the Research Ethics committee of Guangzhou Medical University (GY2022-123) and were performed per the NIH Guide for the Care and Use of Laboratory Animals. Ovariectomies (OVX) were performed, and the mice were recovered for 1 week. Subsequently, pellets containing either control or 17β-estradiol (E2, 0.36 mg, 90-day release, Innovative Research of America, FL, USA) were subcutaneously implanted. Recombinant FSH (3 IU/day, Gonal-F, Serono, Sweden) was mixed in physiological saline and administered daily via subcutaneous injection. The mice were kept on a 12-h light/dark cycle in air-conditioned rooms with free access to high-fat rodent diet containing 1.25% cholesterol (no. D12108C, Research Diets, Inc., USA) and water to induce atherosclerosis. Euthanasia was performed using 2–3% isoflurane inhalation followed by cardiac exsanguination or cervical dislocation when appropriate. FSH and E2 concentrations were ascertained with an enzyme-linked immunosorbent assay (ELISA) kit per the supplier’s protocols (E2: no. 501890, Cayman, Michigan, USA; FSH: no. E-EL-M0511c, Elabscience Biotechnology Co., Ltd, China). All procedures complied with the animal welfare guidelines of Guangzhou Medical University. The mouse whole aortas, aortic root, and brachiocephalic artery trunk were isolated and processed for subsequent analysis.

### Cell culture

Human umbilical vascular endothelial cells (HUVECs) were procured from umbilical cords obtained from Guangzhou First People’s Hospital (Guangdong, China). The isolation of HUVECs was performed per the protocol established by Simoncini et al. [24]. Specifically, after the umbilical vein was rinsed twice with phosphate-buffered saline (PBS), the umbilical vein endothelial cells were digested with 20 mL PBS containing 0.1% type 2 collagenase (no. C2-28, Sigma) and 1% bovine serum albumin (BSA), maintained at 37 °C for 18 min. Then, the digestion was terminated with endothelial cell medium, and the cells were procured by centrifugation at 1000 rpm for 5 min. Cells were cultured in endothelial cell medium (ECM) comprising 5% FBS (no. 1001-prf, ScienCell, California, USA). For all experiments, HUVECs between passages 2 and 5 were utilized.

### Western blot (WB)

Cell lysates were processed for WB analysis using the GE Amersham Imager600. Protein levels in the lysates were ascertained utilizing the Pierce BCA Protein Assay Kit (Thermo Fisher Scientific). Identical quantities of protein (10–15 μg) underwent separation via sodium dodecyl-sulfate polyacrylamide gel electrophoresis (SDS-PAGE), transferred to polyvinylidene fluoride (PVDF) membranes (Millpore), and sealed. The membranes underwent incubation with specific primary antibodies targeting CD31, vWF, VE-cadherin, SM22α, α-SMA, FOXM1, SNAIL, SLUG, TWIST1, FAP, ALKBH5, phosphorylated and total CREB, and GAPDH. Horseradish peroxidase (HRP)-conjugated secondary antibodies were used for detection.

### Quantitative real-time PCR

Total cellular RNA was procured employing an RNA purification kit (no. AG21022, Accurate Biology, China). Reverse transcription was carried out on 500 ng of RNA with the Evo M-MLV Reverse Transcriptase kit (no. AG11706, Accurate Biology, China) per the supplier’s protocols. Quantitative real-time polymerase chain reaction (qRT-PCR) was then executed utilizing SYBR Green Premix Pro Taq HS qPCR Kit (no. AG11701, Accurate Biology, China) on a CFX96 touch real-time system (Bio-Rad), with relative gene expression computed via the 2^−ΔΔCt^ technique and *GAPDH* (human/mouse) serving as the internal control. All primer sequences are depicted in Supplementary Material Table S2.

### Gene silencing

A smart pool of target gene siRNA (containing three target-specific 19–25 nt siRNA) and nontargeting control siRNA were acquired from Santa Cruz (Dallas, Texas, USA). HUVECs underwent transfection with either control siRNA or the target gene siRNA using Lipofectamine RNAiMAX (Thermo Fisher Scientific, USA) per the supplier’s protocols. The infection efficiency was confirmed by qRT-PCR or WB, and the cells were utilized for subsequent experiments 48 h posttransfection.

### Migration assay

HUVEC migration was assessed utilizing a Culture-Insert 4 Well (no. 80466, ibidi, Munich, Germany) in a 35 mm dish. Briefly, 50,000 cells in 100 μL of basal ECM medium (0.5% FBS) were seeded into the four chambers of the Culture-Insert 4 Well and allowed to reach 100% confluence. The Culture-Insert was gently lifted away, and the initial scratch area was imaged and recorded as T0. After 24 h of incubation at 37 °C, the scratch area was photographed and marked as T24. The extent of cell migration and the scratch area in each group were quantified using NIH Image J software.

### Plasmid transfection

All plasmids were sourced from Obio Technology Corp., Ltd. (Shanghai, China). HUVECs were kept in ECM comprising 5% FBS. For transfection studies, cells were placed in an antibiotic-free medium. Transient plasmid transfection was executed utilizing Lipofectamine 3000 reagent (Thermo Fisher Scientific, USA) per the supplier’s protocols. All cells were cultured at 37 °C in a 5% CO_2_ atmosphere.

### Luciferase reporter assay

All luciferase reporter plasmids were sourced from Obio Technology Corp., Ltd. (Shanghai, China). HUVECs were placed at 1 × 10^5^ cells/mL in 24-well plates (Corning, USA) and grown to 70% confluency. The cells were then cotransfected with 1 μg of luciferase plasmid and 0.5 μg of pRL-null *Renilla* plasmid, which served as an internal control, in serum-free ECM utilizing Lipofectamine 3000 reagent (Thermo Fisher Scientific, USA) per the supplier’s protocols. Posttransfection, the cells underwent lysis, and the lysed material was gathered through centrifugation at 10,000 rpm for 1 min. The luciferase signals were detected utilizing the Dual-Luciferase Reporter Assay System (Promega, USA) per the supplier’s protocols. The relative light units of firefly luciferase were standardized against *Renilla* luciferase activity to obtain a normalized luciferase measurement.

### Immunohistochemistry and immunofluorescence

Lipid deposition was assessed via ORO staining. In brief, frozen aortic sections underwent PBS washing to eliminate the optimal cutting temperature (OCT) compound, followed by incubation with 60% isopropanol. Sections were then stained with 2% (*w*/*v*) ORO solution and subjected to hematoxylin counterstaining. Images were captured utilizing a phase-contrast microscope.

For immunofluorescence, frozen sections underwent PBS washing to eliminate the OCT compound and were treated with 0.1% Triton X-100 for 15 min to achieve permeabilization. The arterial sections were then subjected to blocking and exposed to primary antibodies targeting VE-cadherin and vimentin overnight in a humidified chamber at 4 °C. Following the washing steps, the sections were exposed to fluorescence-conjugated secondary antibodies for 1 h at ambient conditions, then mounted employing glycerol mounting medium containing 4′,6-diamidino-2-phenylindole (DAPI). Images were captured utilizing a Leica DLS inverted confocal microscope, and fluorescence analysis was executed using Image-Pro Plus software.

### RNA immunoprecipitation

RNA immunoprecipitation was executed utilizing the Magna RIP RNA-Binding Protein Immunoprecipitation Kit (Millipore) per the supplier’s protocol. In short, magnetic beads covered with 5 μg of antibodies specific to mouse IgG, FLAG (Sigma Aldrich), or Hu antigen R (HuR) (Santa Cruz) underwent incubation with previously frozen cell lysates throughout the night at 4 °C. Subsequently, the RNA–protein complexes were gathered, underwent six washing cycles, and were processed with proteinase K digestion before RNA extraction employing TRIzol. The comparative association between protein and RNA underwent quantification through qPCR and was standardized to the input. IgG functioned as a negative control.

### Lentiviral transduction for cells

Lentiviral vectors expressing nontargeting control shRNA and two different ALKBH5 (NM_017758) shRNAs were acquired from Obio Technology Corp., Ltd. (Shanghai, China). HUVECs were then transduced with these lentiviruses in the presence of polybrene (5 mg/mL, Sigma). After 24 h of transduction, the cells experienced selection through exposure to 2 μg/mL puromycin for 1 day.

### MeRIP assay

The MeRIP assay was executed utilizing Magna MeRIP™ m6A Kit (Millipore). Briefly, 300 µg total RNA was purified from cells, and one-tenth of the total RNA was utilized as the input control. Magna ChIP Protein A/G Magnetic Beads were prewashed and incubated with an anti-m6A antibody for 30 min at room temperature. Next, 200 µg RNA was introduced to the beads–antibody complex and subjected to rotation for 2 h at 4 °C. After adequate washing, immunoprecipitated RNA was eluted in an elution buffer with continuous shaking at 4 °C for 1 h. The eluted RNA was purified by the RNeasy mini kit, and m6A enrichment RNA was determined by qPCR analysis.

### Chromatin immunoprecipitation assays

Chromatin immunoprecipitation assays were executed utilizing the Chromatin Immunoprecipitation (ChIP) Assay Kit (Millipore) per the supplier’s protocol. Briefly, the cells following specific treatment underwent cross-linking with 1% formaldehyde at ambient conditions for 10 min, succeeded by quenching using 125 mM glycine and two rinses with cold PBS. The cells underwent lysis using 100 μL 1% SDS lysis buffer and fragmentation through sonication. The resulting sonicated cell supernatant underwent a ninefold dilution (720 μL) with ChIP dilution buffer, and 20 μL was designated as input. Following overnight incubation at 4 °C with p-CREB (Ser129/133) antibody (ThernoFisher) or normal IgG (negative control) to pull down cross-linked proteins and DNA, the cross links (both input and immunoprecipitated group) were reversed, and DNA (included immunoprecipitated and input group) was isolated through phenol/chloroform extraction. The obtained DNA was subsequently dissolved in 20 μL PCR-grade water. PCR amplification (35 cycles) was performed using 2 μL of input or immunoprecipitated DNA as templates with promoter primers (Supplementary Table S2). The ChIP-PCR products underwent separation on a 1% agarose gel containing ethidium bromide (EB). The enrichment of specific promoter regions post-IP was ascertained as fold induction compared with IgG.

### Statistical analysis

The results are denoted as mean ± standard deviation (SD). Statistical analyses were conducted utilizing GraphPad Prism software (GraphPad Software, Inc.). Comparisons between paired groups were examined through Student’s *t*-test. For multiple group comparisons, one/two-way analysis of variance (ANOVA) with a subsequent appropriate post hoc test was employed. Statistical significance was set at *p* < 0.05.

## Results

### FSH enhances EndMT in atherosclerotic lesions and promotes EndMT in vivo and in vitro

**Fig. 1:**
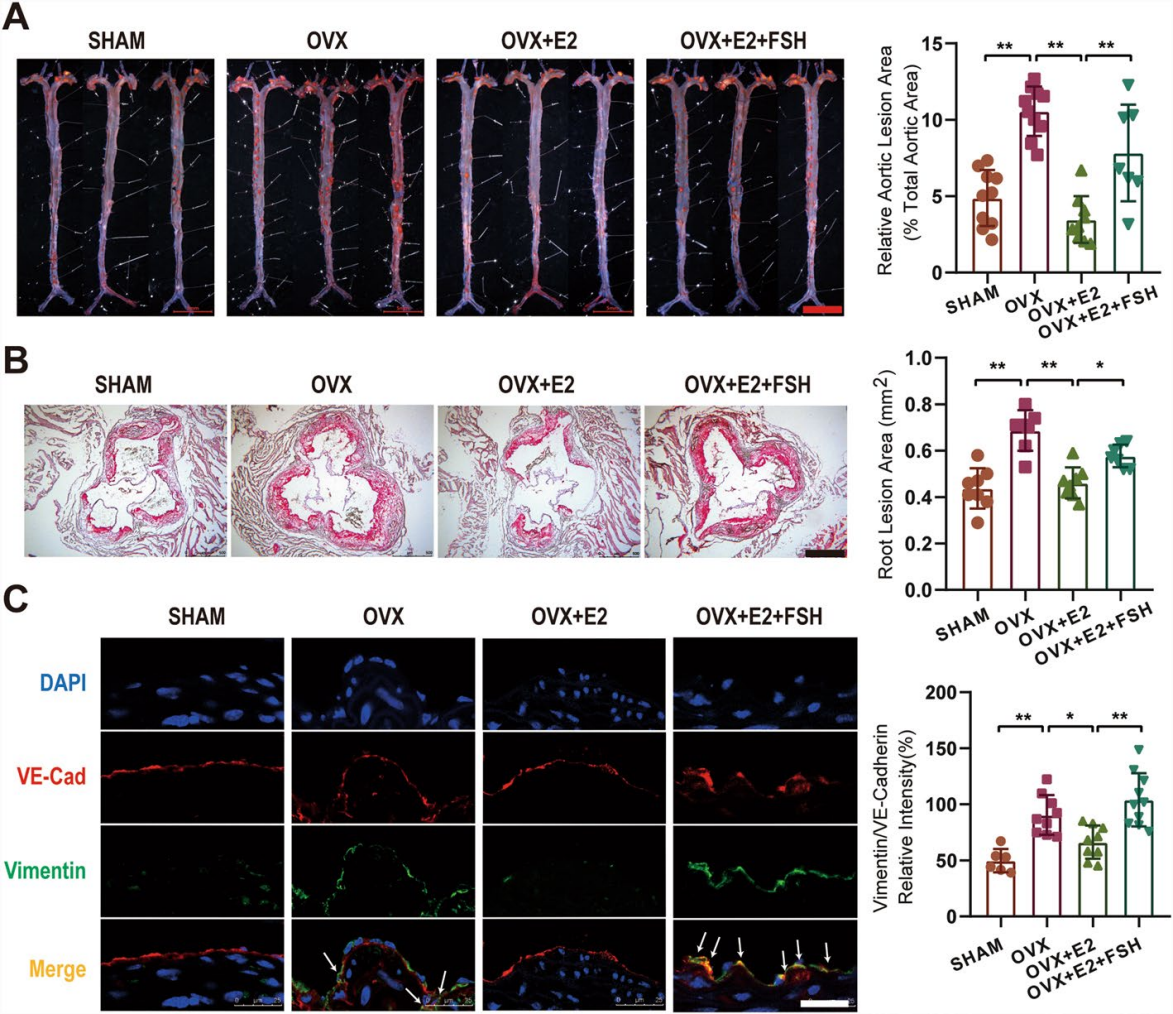
FSH promotes atherosclerotic plaque formation and EndMT in vivo. *Apolipoprotein E*-deficient (*ApoE*^−/−^) mice were fed an atherogenic diet for 12 weeks and treated as indicated. Whole aortas (**A**) and aortic roots (**B**) underwent Oil Red O staining, and atherosclerotic lesions were evaluated by calculating the surface area of Oil Red O–positive lesions in en face preparations. Statistical analysis was executed using one-way ANOVA with Sidak’s multiple comparisons test, with *n* = 6–10; **p < 0.01, *p < 0.05. Brachiocephalic artery trunk immunofluorescence staining was conducted to examine EndMT across treatment groups, using VE-Cadherin (VE-Cad) in red, vimentin in green, and DAPI in blue; white arrows indicate sites of EndMT occurrence (**C**). Scale bars: 5 mm (**A**), 500 μm (**B**), and 25 μm (**C**)

Previous findings have demonstrated that FSH promotes atherosclerotic lesion formation [9]. To explore the role of FSH in regulating EndMT and atherosclerotic plaque instability, ovariectomy (OVX) was performed on high-fat diet-fed *ApoE*^−/−^ mice, followed by estrogen and FSH supplementation. Oil Red O staining of the aorta and aortic root demonstrated a substantial elevation in plaque area in OVX mice (aorta: 10.56 ± 1.62%; aortic root: 0.69 ± 0.09 mm^2^) compared with the SHAM control (aorta: 4.88 ± 1.83%; aortic root: 0.44 ± 0.09 mm^2^). While estrogen supplementation reduced plaque formation (aorta: 3.47 ± 1.53%; aortic root: 0.46 ± 0.07 mm^2^), FSH injection counteracted this effect, leading to an increased plaque area (aorta: 7.83 ± 3.16%; aortic root: 0.58 ± 0.05 mm^2^) (Fig. 1A, B). As previously noted [25], VE-Cadherin and vimentin serve as markers for endothelial and mesenchymal cells, respectively. Immunofluorescence staining indicated that estrogen mitigated EndMT in vivo (vimentin/VE-Cadherin%: 66.27 ± 14.78%) compared with the OVX group (vimentin/VE-Cadherin%: 90.46 ± 17.66%), whereas FSH negated this protective effect (vimentin/VE-Cadherin%: 104.07 ± 23.68%) (Fig. 1C).

**Fig. 2:**
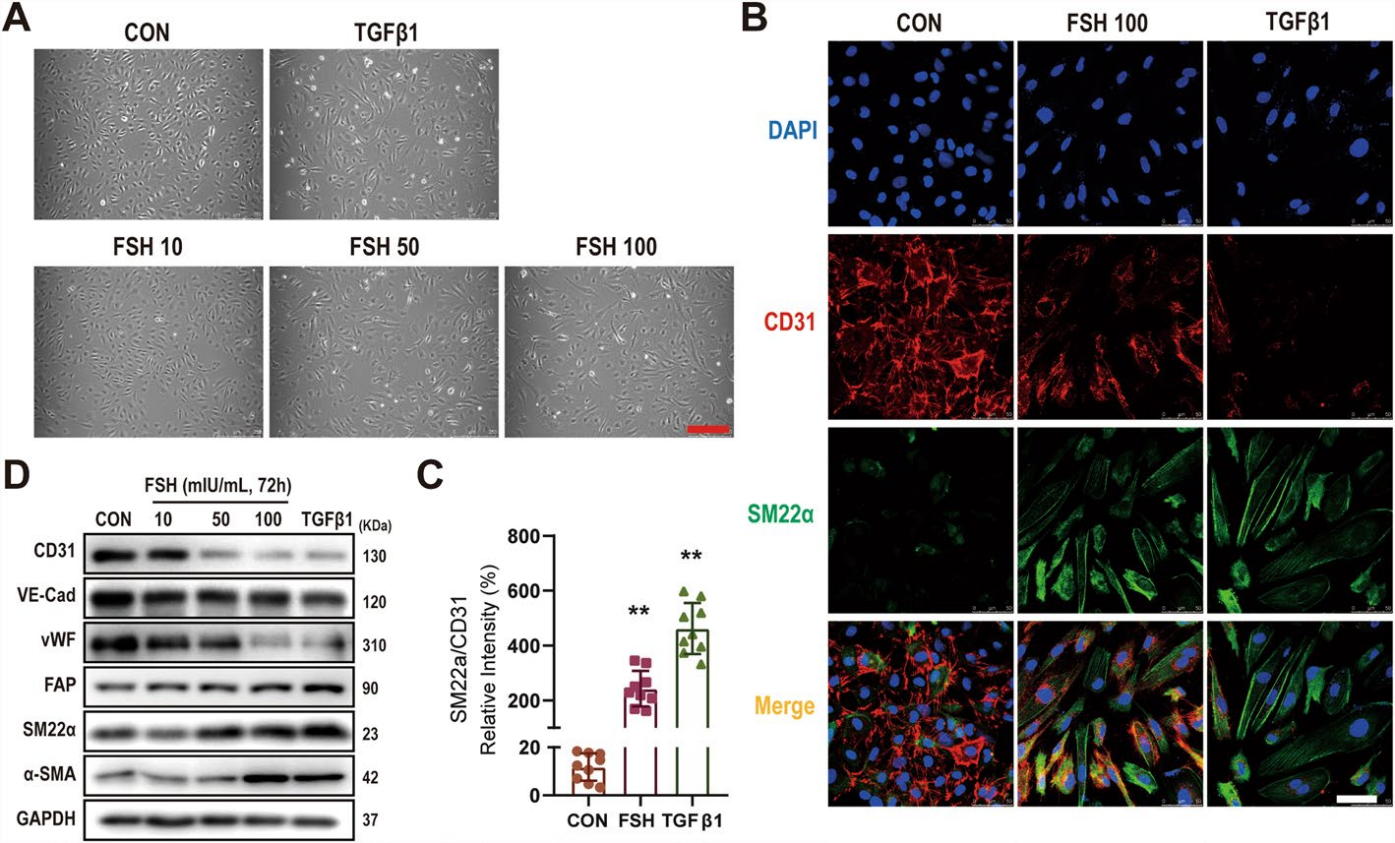
FSH promotes EndMT in HUVECs. Morphological changes in HUVECs were observed following treatment with FSH (10–100 mIU/mL) and TGFβ1 (10 ng/mL) as a positive control for 3 days (**A**). The expression of endothelial markers CD31, VE-cadherin (VE-Cad), and vWF, along with mesenchymal markers FAP, SM22α, and α-SMA, was assessed by immunofluorescence staining (**B**) and WB analysis (**D**). **C** Quantification of protein expression was performed by analyzing fluorescence intensity values (CON: 11.78 ± 5.73%; FSH: 242.48 ± 64.92%; TGFβ: 462.33 ± 93.11%). Statistical analysis was conducted using one-way ANOVA with Dunnett’s multiple comparisons test (**C**), *n* ≥ 3, **p < 0.01. Scale bars: 250 μm (**A**), 50 μm (**B**)

The role of FSH in EndMT was further investigated in vitro. Transforming growth factor beta 1 (TGFβ1), a well-established inducer of EndMT, served as a positive control [25]. As depicted in Fig. 2A, FSH induced morphological changes in HUVECs, transitioning from their original oval shapes to elongated spindle-like forms, albeit less potently than TGFβ1 (Fig. 2A). High concentrations of FSH (50–100 mIU/mL) markedly downregulated the expression of endothelial markers CD31, VE-cadherin (VE-Cad), and vWF, while upregulating mesenchymal markers FAP, SM22α, and α-SMA (Fig. 2B–D). These results collectively suggest that FSH promotes EndMT both in vivo and in vitro.

### FSH promotes EndMT via transcriptional factor FOXM1

**Fig. 3:**
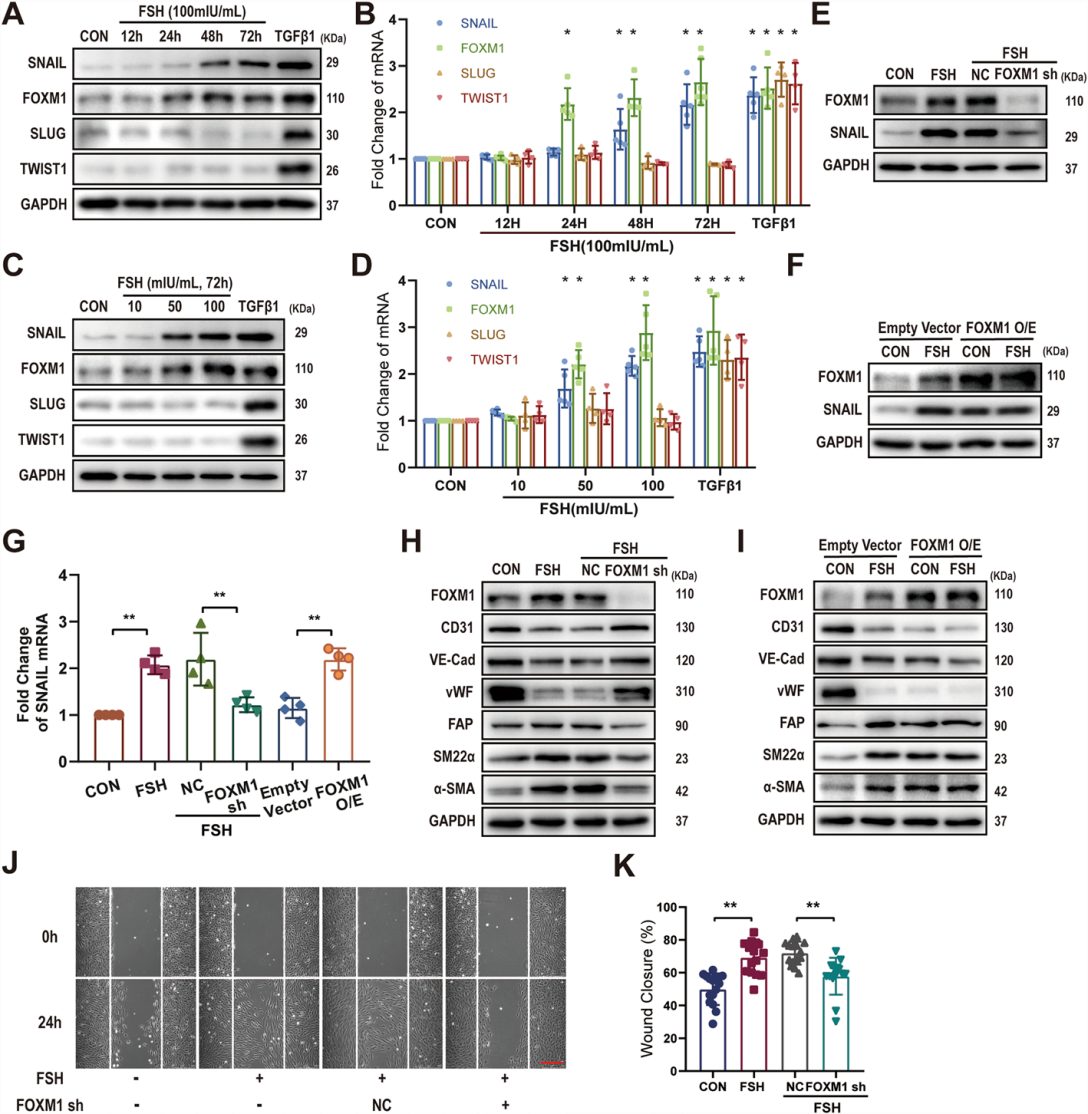
FSH promotes EndMT via the transcriptional factor FOXM1. The effects of FSH on the protein levels of EndMT-related transcription factors were assessed by WB (**A** and **C**), while changes in transcription factor mRNA levels were analyzed by RT-qPCR (**B** and **D**) in HUVECs. HUVECs were transfected with *FOXM1* shRNA or FOXM1 overexpression (FOXM1 O/E) constructs and exposed to FSH (100 mIU/mL) for 48 h. FOXM1 and SNAIL protein levels were determined by WB (**E** and **F**), and SNAIL mRNA expression levels were measured by RT-PCR (**G**). Additionally, HUVECs transfected with *FOXM1* shRNA or *FOXM1* O/E and treated with FSH (100 mIU/mL) for 72 h were examined for the expression of EndMT-related proteins by WB (**H** and **I**). **J** Following *FOXM1* shRNA transfection, HUVECs were treated with FSH (100 mIU/mL) for 72 h, and cell migration was evaluated using a wound-healing assay over 24 h. **K** Quantification of the wound-healing assay. Statistical analysis was executed utilizing two-way ANOVA with Dunnett’s multiple comparisons test (**B** and **D**). Data from RT-PCR and wound-healing assays were examined utilizing one-way ANOVA with Sidak’s multiple comparisons test (**G** and **K**). *n* ≥ 3, *p < 0.05, **p < 0.01. Scale bars: 250 μm (**J**)

Previous studies have identified transcription factors such as SNAIL, SLUG, TWIST1, and FOXM1 as key regulators of EndMT in vascular endothelial cells [22, 25]. To assess the impact of FSH on these molecules, their expression levels were analyzed. Treatment with high concentrations of FSH (50–100 mIU/mL) for 48–72 h markedly upregulated both protein and mRNA levels of FOXM1 (fold change: 2.32 ± 0.40 to 2.88 ± 0.59 in HUVECs) and SNAIL (fold change: 1.64 ± 0.44 to 2.17 ± 0.21 in HUVECs), while SLUG and TWIST1 expression remained unchanged in HUVECs (Fig. 3A–D).

FOXM1 is a well-established transcription factor that binds to the promoter region of *SNAIL*, thereby promoting its expression [22]. To elucidate their relationship, FOXM1 expression was either silenced or overexpressed in HUVECs. Silencing FOXM1 abolished the FSH-induced upregulation of *SNAIL* mRNA (fold change: from 2.19 ± 0.57 to 1.22 ± 0.16 in HUVECs) and protein levels (Fig. 3E and G). Conversely, overexpression of FOXM1 markedly enhanced SNAIL expression (fold change: from 1.15 ± 0.22 to 2.19 ± 0.24 in HUVECs), overshadowing the effect of FSH (fold change: 2.08 ± 0.20 in HUVECs) (Fig. 3F and G).

The role of FOXM1 in FSH-promoted EndMT was further confirmed. When FOXM1 was silenced, FSH lost its ability to decrease endothelial marker levels and increase mesenchymal marker levels (Fig. 3H). In contrast, FOXM1 overexpression induced the classic EndMT marker expression pattern, with FSH providing no additional effect under these conditions (Fig. 3I). EndMT is associated with enhanced cellular migratory ability [25]. Consistent with this, FSH promoted HUVECs migration (wound closure%: from 49.91 ± 9.59% to 69.23 ± 9.97%), but this effect was blocked by FOXM1 knockdown (wound closure%: from 71.95 ± 6.99% to 57.82 ± 11.30%) (Fig. 3J, K). Collectively, these observations suggest that FOXM1 mediates FSH’s influence on EndMT in vascular endothelial cells.

### FSH increases FOXM1 expression through ALKBH5-mediated m6A demethylation

**Fig. 4:**
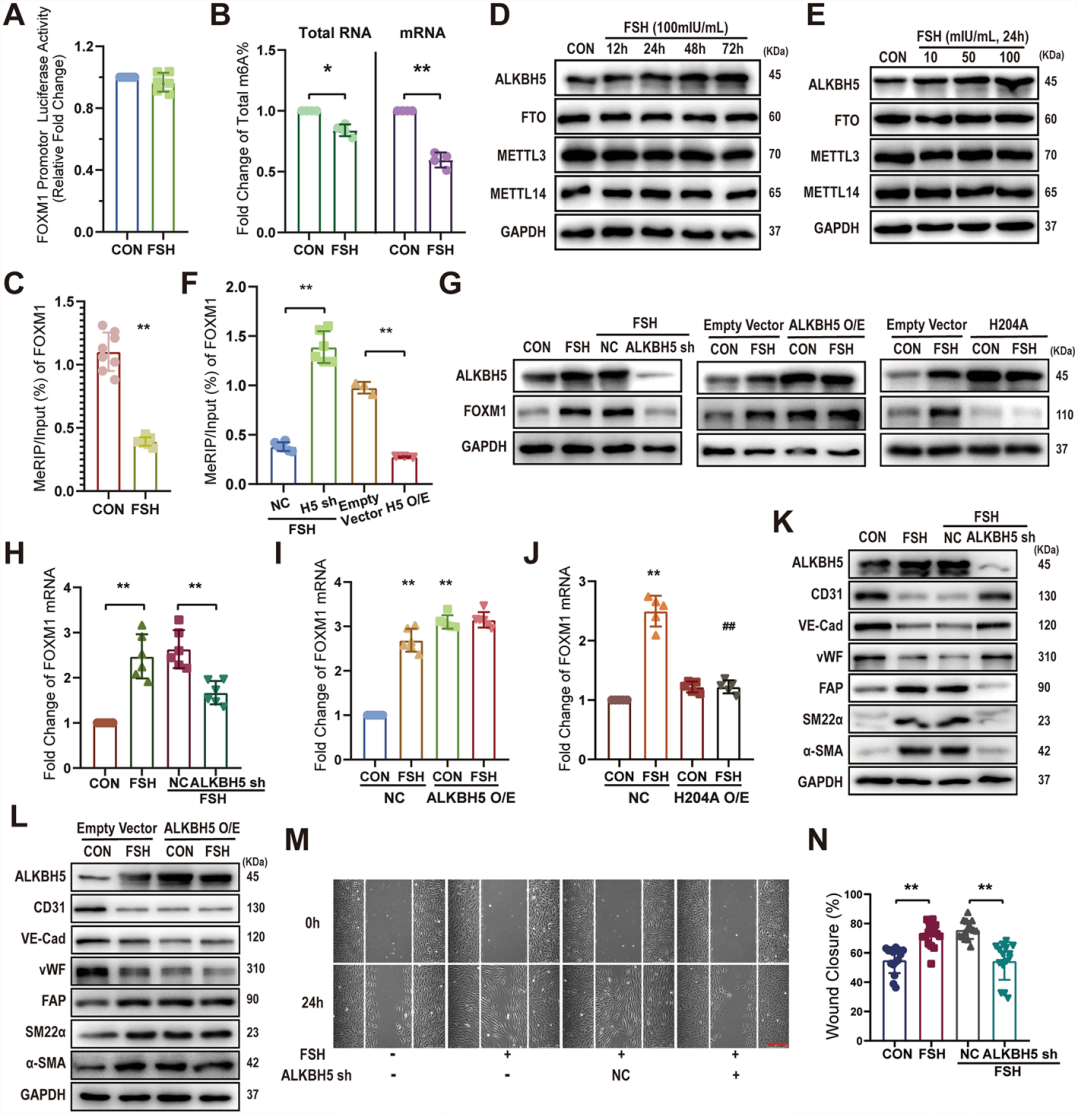
FSH increases FOXM1 expression through ALKBH5-mediated m6A demethylation. HUVECs were treated with FSH (100 mIU/mL) for 24 h, and the relative activity of the *FOXM1* promoter firefly luciferase reporter (1 μg) was measured (**A**). The proportion of m6A in total RNA and total mRNA was assessed by colorimetry (**B**), and *FOXM1* mRNA m6A levels were analyzed by MeRIP (**C**). HUVECs were treated with varying concentrations of FSH (10–100 mIU/mL) for different durations (12–72 h), and protein expression levels of ALKBH5, FTO, METTL3, and METTL4 were detected by WB (**D**, **E**). HUVECs were transfected with *ALKBH5* shRNA or *ALKBH5* overexpression (O/E) constructs and treated with FSH (100 mIU/mL) for 24 h, after which *FOXM1* mRNA m6A levels were detected by MeRIP (**F**). Following transfection with *ALKBH*5 shRNA, *ALKBH5* O/E, or the *ALKBH5* H204A mutant, HUVECs were treated with FSH (100 mIU/mL) for 24 h, and the indicated protein expressions were measured by WB (**G**), with *FOXM1* mRNA levels determined by qRT-PCR (**H**–**J**). HUVECs transfected with *ALKBH5* shRNA or *ALKBH5* O/E were treated with FSH (100 mIU/mL) for 72 h, and EndMT-related protein expression was analyzed by WB (**K**, **L**). **M** Following *ALKBH5* shRNA transfection, HUVECs were treated with FSH (100 mIU/mL) for 72 h, and cell migration was assessed using a wound-healing assay over 24 h. **N** Quantification of the wound-healing assay. Data were analyzed using an unpaired *t*-test (**A**, **C**) and one-way ANOVA with Sidak’s multiple comparisons test (**F**, **H**, **I**, **J**, **N**). Statistical analysis was conducted using two-way ANOVA with Sidak’s multiple comparisons test (**B**). *n* ≥ 3, *p < 0.05, **p < 0.01. Scale bars: 250 μm (**M**)

As shown in Fig. 3, FSH increased both FOXM1 mRNA and protein expression. However, the dual-luciferase reporter assay revealed that FSH did not enhance *FOXM1* gene promoter activity, suggesting that the upregulation of FOXM1 by FSH occurs at the posttranscriptional level (Fig. 4A). m6A modification, the most prevalent internal modification of mRNAs, influences various aspects of RNA expression and translation [26]. Indeed, m6A quantification assays demonstrated that FSH reduced m6A levels in total RNA (fold change: 0.84 ± 0.05), total mRNA (fold change: 0.60 ± 0.06), and, specifically, *FOXM1* mRNA (MeRIP/Input %: from 1.10 ± 0.15% to 0.39 ± 0.03%) in HUVECs (Fig. 4B, C).

The dynamic regulation of mRNA m6A levels is governed by m6A methylases and demethylases. FSH was found to enhance the expression of the demethylase ALKBH5 in a time- and concentration-dependent manner, without affecting the expression of the demethylase FTO or the methylases METTL3 and METTL14 (Fig. 4D, E). Silencing ALKBH5 prevented FSH from decreasing *FOXM1* mRNA m6A levels (MeRIP/Input%: from 0.38 ± 0.04% to 1.39 ± 0.16%) and from increasing *FOXM1* mRNA (fold change: form 2.64 ± 0.42 to 1.67 ± 0.26) and protein expression (Fig. 4F, H). Conversely, upregulation of wild-type (WT) ALKBH5, but not the catalytically inactive mutant ALKBH5 H204A, markedly elevated *FOXM1* mRNA (fold change: 3.10 ± 0.15) and protein levels (Fig. 4G, I, J). These results suggest that FSH increases *FOXM1* expression through ALKBH5-mediated m6A demethylation.

The role of ALKBH5 in FSH-promoted EndMT was further examined. FSH diminished endothelial markers (CD31, VE-cadherin, vWF) levels and elevated mesenchymal marker levels (SM22α, FAP, α-SMA), effects that were abolished when ALKBH5 was silenced (Fig. 4K). Consistently, ALKBH5 overexpression strongly induced the typical expression pattern of these markers, regardless of FSH presence (Fig. 4L). Additionally, the ability of FSH to promote HUVECs migration was also blocked following ALKBH5 knockdown (wound closure%: from 72.06 ± 7.94% to 54.55 ± 12.95%) (Fig. 4M, N).

### ALKBH5 stabilizes *FOXM1* mRNA via RNA binding protein HuR

**Fig. 5:**
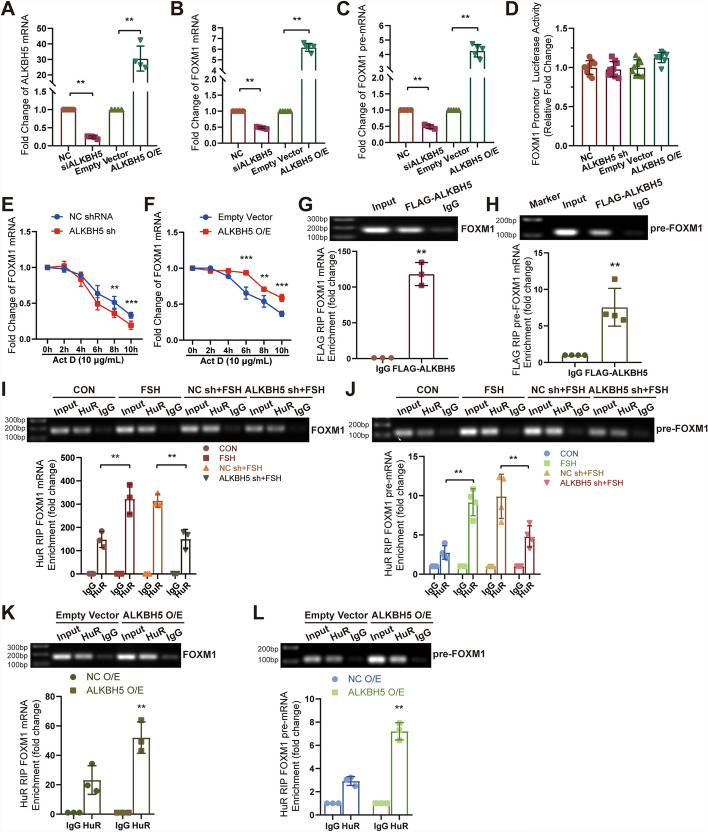
ALKBH5 stabilizes *FOXM1* mRNA via the RNA binding protein HuR. HUVECs were transfected with either ALKBH5 siRNA (30 nM) or an overexpression plasmid (1 μg), and the levels of indicated mature or pre-mRNA were assessed by qRT-PCR (**A**–**C**). HUVECs were infected with lentiviruses containing* ALKBH5* shRNA, *ALKBH5* plasmid, or a negative control plasmid to generate *ALKBH*5-silenced or overexpressing stable cell lines. The relative activity of the *FOXM1* promoter firefly luciferase reporter (1 μg) was measured (**D**). RNA from HUVECs treated with 10 μg/mL actinomycin D (Act D) for 2 to 10 h was collected and analyzed by qRT-PCR (**E**, **F**). HUVECs were transfected with exogenous FLAG-tagged ALKBH5, and the enrichment of *FOXM1* mRNA or pre-mRNA with FLAG was measured by RIP followed by qRT-PCR, normalized to input (**G**, **H**). RIP analysis was used to detect the interaction of *FOXM1* pre-mRNA or mature mRNA with HuR in HUVECs treated as indicated. The enrichment of RNA with HuR was quantified by qRT-PCR (**I**–**L**). Statistical analysis was conducted using one-way ANOVA with Sidak’s multiple comparisons test (**A**–**D**), two-way ANOVA with Sidak’s multiple comparisons test (**E**, **F**, **I**–**L**), and unpaired *t*-test (**G**, **H**). *n* ≥ 3, *p < 0.05, **p < 0.01

Transfection of HUVECs with *ALKBH5* siRNA or an encoding plasmid (Fig. 5A) resulted in the downregulation or upregulation of *FOXM1* pre-mRNA (fold change: 0.49 ± 0.06 or 4.26 ± 0.43) and mature mRNA (fold change: 0.48 ± 0.03 or 6.18 ± 0.37), respectively (Fig. 5B, C). To clarify the underlying mechanism, the potential role of ALKBH5 in regulating *FOXM1* gene transcription was first examined. A luciferase reporter assay revealed that neither silencing nor overexpression of ALKBH5 affected the transcriptional activity of the *FOXM1* promoter, indicating a posttranscriptional regulatory mechanism (Fig. 5D). The effect of ALKBH5 on *FOXM1* mRNA degradation was then investigated using the transcription inhibitor actinomycin D (Act D) to block new RNA synthesis and assess the mRNA half-life. The half-life of *FOXM1* mRNA was reduced from 8 to 6 h upon ALKBH5 silencing, whereas it was extended to 10 h following ALKBH5 overexpression (Fig. 5E, F). These observations suggest that ALKBH5 enhances the stability of *FOXM1* mRNA.

ALKBH5 directly interacts with target mRNAs to stabilize them. To verify this interaction, FLAG-tagged ALKBH5 was overexpressed in HUVECs, and RIP assays confirmed cross-linking between ALKBH5 and both *FOXM1* pre-mRNA (enrichment fold change: 7.57 ± 2.58) and mature mRNA (enrichment fold change: 118.22 ± 16.12) (Fig. 5G, H). Furthermore, recent studies have shown that ALKBH5 enhances the binding of HuR protein to *FOXM1* mRNA, thereby promoting its expression [17]. Consistent with these results, FSH was observed to increase the cross-linking of *FOXM1* pre-mRNA and mRNA with HuR protein (enrichment fold change: from 2.75 ± 0.74 to 9.16 ± 1.49 and from 148.01 ± 28.18 to 322.38 ± 51.96), an effect that was diminished when ALKBH5 expression was silenced (enrichment fold change: from 9.92 ± 2.43 to 4.81 ± 1.18 and from 315.30 ± 23.41 to 150.40 ± 33.88) (Fig. 5I, J). Conversely, ALKBH5 overexpression led to increased cross-linking (enrichment fold change: from 2.92 ± 0.31 to 7.21 ± 0.61 and from 23.19 ± 7.95 to 52.10 ± 8.74) (Fig. 5K, L). These results suggest that FSH, through ALKBH5, enhances the interaction between HuR protein and *FOXM1* pre-mRNA and mRNA, thereby stabilizing FOXM1 RNA and increasing its expression.

### FSH upregulated *ALKBH5* mRNA expression via CREB signaling

**Fig. 6:**
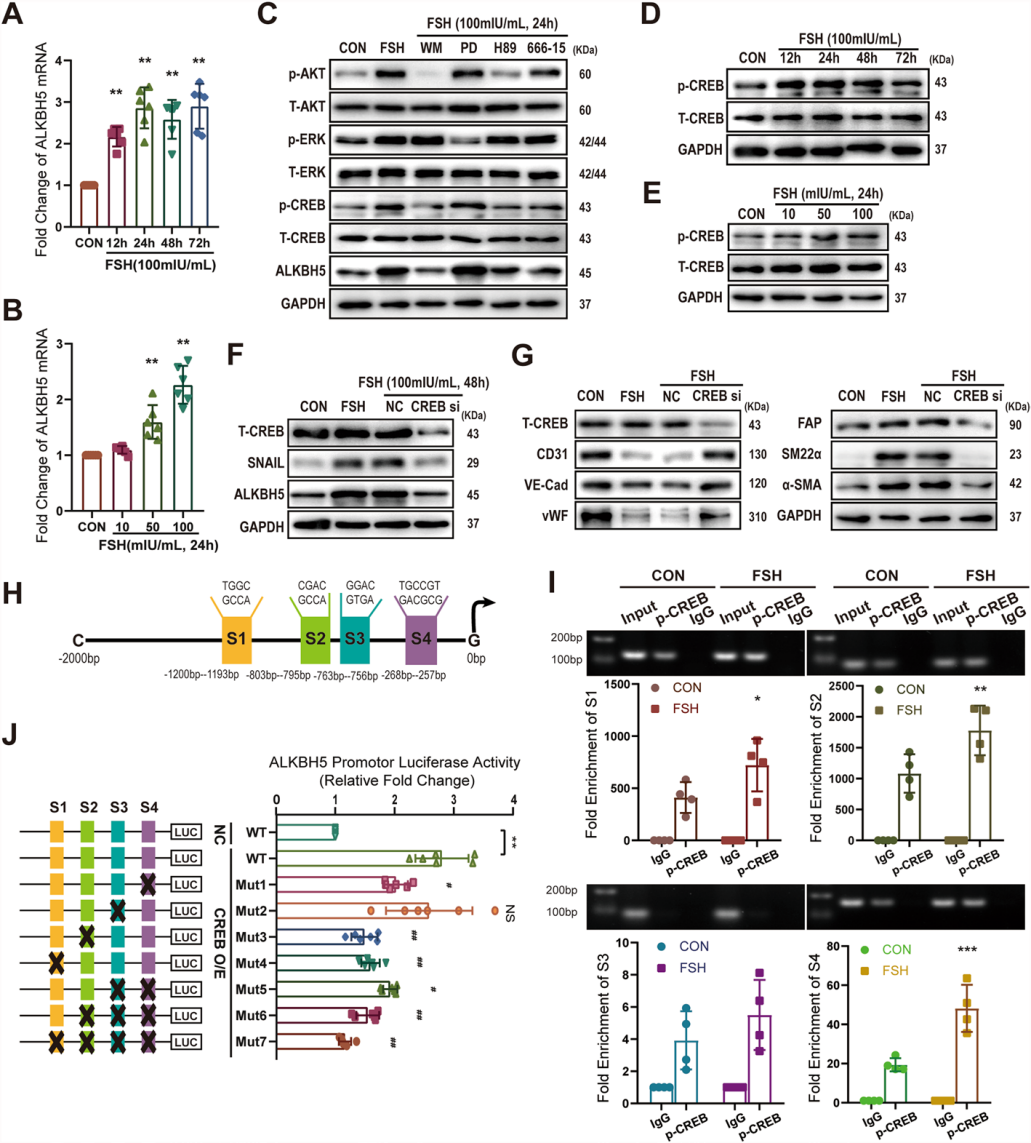
FSH upregulates *ALKBH5* mRNA expression via CREB signaling. HUVECs were treated with varying concentrations of FSH for different time intervals, and *ALKBH5* mRNA levels were assessed by qRT-PCR (**A**, **B**). HUVECs were treated with PI3K inhibitor wortmannin (WM, 1 μM), MEK inhibitor PD98059 (PD, 20 μM), PKA inhibitor H89 (10 μM), or CREB inhibitor 666–15 (10 μM), followed by treatment with FSH (100 mIU/mL) for 24 h. Protein expression was analyzed by WB (**C**). HUVECs were treated with different concentrations of FSH (10–100 mIU/mL) for varying durations (12–72 h), and the protein expression of total CREB and phosphorylation of CREB at Ser129/133 was detected by WB (**D**, **E**). HUVECs transfected with *CREB* siRNA were treated with FSH (100 mIU/mL) for 48–72 h, and protein expression was analyzed by WB (**F**, **G**). A schematic overview depicts the predicted p-CREB S129/133 binding sites within the *ALKBH5* promoter region, with vertical bars indicating binding sites (**H**). HUVECs were treated with or without FSH (100 mIU/mL) for 24 h, and ChIP-qPCR confirmed the binding of p-CREB to the *ALKBH5* promoter (**I**). A schematic diagram illustrates deletion plasmids containing different *ALKBH5* promoter fragments upstream of the firefly luciferase reporter gene in the pGL4.10 vector (left). Following transfection of deletion plasmids and CREB overexpression (CREB O/E) or negative control plasmid (NC) into 293 T cells for 24 h, luciferase activity was measured (**J**, **p < 0.01 versus NC + WT, #*p* < 0.05, ##*p* < 0.01 versus CREB O/E + WT). Statistical analysis was conducted using one-way ANOVA with Dunnett’s multiple comparisons test (**A**, **B**) and Sidak’s multiple comparisons test (**J**). Data were analyzed by two-way ANOVA with Sidak’s multiple comparisons test (**I**). *n* ≥ 3, *p < 0.05, **p < 0.01

As previously mentioned, FSH increases ALKBH5 protein expression. Consistent with this, FSH also upregulated *ALKBH5* mRNA expression in a time- and concentration-dependent manner (range of fold change: from 1.60 ± 0.30 to 2.90 ± 0.54 in HUVECs) (Fig. 6A, B). Traditionally, FSH binds to the FSH receptor (FSHR) and couples with the Gαs subunit, triggering downstream signaling pathways including PI3K/Akt, PKA/CREB, and ERK1/2 [27]. To investigate the roles of these pathways in FSH-induced ALKBH5 expression, HUVECs were treated with specific inhibitors: wortmannin (WM) for PI3K, H89 for PKA, 666-15 for CREB, and PD98059 (PD) for ERK1/2. The results showed that inhibitors targeting PI3K, PKA, and CREB significantly reduced FSH-induced ALKBH5 expression at both the protein and mRNA levels. Specifically, the fold change in ALKBH5 expression decreased from 2.25 ± 0.34 to 1.13 ± 0.27 with WM (PI3K inhibitor), to 1.26 ± 0.23 with H89 (PKA inhibitor), and to 1.38 ± 0.29 with 666–15 (CREB inhibitor) (Fig. 6C and Supplementary Fig. S1C). In contrast, the ERK1/2 inhibitor PD98059 had no effect on ALKBH5 expression. Furthermore, FSH was shown to activate the phosphorylation of Akt, ERK, and CREB (Fig. 6C). Notably, the PI3K inhibitor WM attenuated FSH-induced CREB activation, while the CREB inhibitor did not affect FSH-induced Akt phosphorylation (Fig. 6C), indicating that PI3K acts upstream of CREB the FSH signaling cascade, regulating ALKBH5 expression through this hierarchical pathway. In summary, these findings indicate that FSH upregulates ALKBH5 expression primarily through the PI3K/Akt and PKA/CREB pathways, with PI3K positioned upstream of CREB, while ERK1/2 signaling does not appear to play a significant role in this process.

CREB is a well-known transcription factor that regulates various target genes when phosphorylated. FSH was observed to upregulate CREB phosphorylation at Ser129/133 in a time- and concentration-dependent manner (Fig. 6D, E). Knockdown of *CREB* using siRNA abolished FSH-induced upregulation of Snail, ALKBH5 expression, and EndMT marker protein expression (Fig. 6F, G), underscoring the pivotal role of CREB in regulating ALKBH5 expression. Using JASPAR prediction, four potential CREB binding sites (S1–S4) were identified in the *ALKBH5* promoter region (Fig. 6H). Chromatin immunoprecipitation revealed that under normal conditions, phosphorylated CREB (p-CREB Ser129/133) strongly cross-linked with the S1, S2, and S4 sites of the *ALKBH5* promoter, while binding with S3 was weak (fold enrichment: 411.26 ± 147.90 (S1), 1083.23 ± 311.49 (S2), 3.92 ± 1.81 (S3), and 19.40 ± 3.84 (S4)) (Fig. 6I). FSH treatment markedly enhanced p-CREB Ser129/133 binding to S1, S2, and S4 (fold enrichment: 722.00 ± 251.53 (S1), 1778.59 ± 401.47 (S2), and 48.18 ± 12.03 (S4)) (Fig. 6I). To assess the transcriptional activity of these binding sites, wild-type (WT) and mutated plasmids containing luciferase reporter genes were transfected into 293 T cells. The results showed that WT CREB plasmid transfection markedly promoted *ALKBH5* transcriptional activity (fold change: 2.80 ± 0.45). Compared with WT, only the mut-2 site showed no significant difference, while mutations in the other sites resulted in a significant decrease in transcriptional activity [fold change: from 2.80 ± 0.45 (WT) to 2.03 ± 0.20 (Mut1), to 2.58 ± 0.73 (Mut2), to 1.49 ± 0.22 (Mut3), to 1.60 ± 0.16 (Mut4), to 1.93 ± 0.12 (Mut5), to 1.55 ± 0.19 (Mut6), to 1.16 ± 0.10 (Mut7)] (Fig. 6J). These results indicate that FSH promotes *ALKBH5* gene transcription by enhancing the binding of phosphorylated CREB to the S1, S2, and S4 sites in the *ALKBH5* promoter region.

## Discussion

The decline in ovarian function and the associated changes in serum hormone levels are significant risk factors for the increased incidence of cardiovascular disease (CVD) in women during and after menopause. For example, stroke is significantly less common in premenopausal women compared with young men, but its incidence doubles in women during the menopausal transition (ages 45–54 years), with more severe outcomes over time [28]. This increased risk and poor prognosis in postmenopausal women are often attributed to decreased levels of estrogen (E2). However, studies suggest that the decline in E2 may not be the sole factor driving this risk. Clinical research has shown that the incidence of CVD begins to rise in women even before estrogen levels decline during the menopausal transition [29]. Furthermore, estrogen supplementation after menopause has not been found to alleviate or improve the occurrence and prognosis of CVD [4].

In addition to decreased estrogen levels, women experiencing ovarian function decline also exhibit a significant rise in follicle-stimulating hormone (FSH) levels, which begins approximately 6 years before the final menstruation and remains elevated after menopause [6]. This elevation in FSH has been implicated in adverse cardiovascular effects. For instance, FSH promotes lipid production and storage, which can be blocked by polyclonal antibodies targeting the FSH β subunit [30]. Clinical data further support this, showing that premenopausal women with serum FSH levels > 7 IU/I have significantly higher total cholesterol compared with those with FSH levels < 7 IU/I [31]. Elevated FSH levels are also associated with vascular inflammation in postmenopausal women [32], implicating FSH as a potential contributor to CVD.

Despite these findings, the precise role and mechanisms of FSH in CVD development remain unclear, sparking considerable research interest. Our previous research found that while E2 supplementation reduced atherosclerotic plaque formation in mice, the introduction of FSH negated this protective effect and increased plaque formation in *ApoE*^−/−^ mice [9]. These results suggest that elevated serum FSH levels may promote atherosclerotic plaque formation. While AS plaque formation provides the pathological foundation for clinical cardiovascular events, it is plaque instability—specifically the vulnerability of the fibrous cap leading to rupture—that primarily drives acute cardiovascular events, particularly acute coronary syndromes [33]. Building on previous studies, the current research explores whether FSH influences AS plaque instability in postmenopausal women.

Plaque instability encompasses both rupture and erosion. Effective lipid control can stabilize most plaques, yet only approximately 5% of plaque ruptures result in clinical events. Clinically, one-third of acute coronary syndromes are triggered by plaque erosion [34], primarily owing to the disruption of the endothelial cell monolayer. Recent advances in endothelium-specific profiling have shifted research focus toward endothelial-mesenchymal transition (EndMT). Clinical samples have shown a positive link between the severity of atherosclerosis and the degree of EndMT [35].

Similar to EMT, EndMT is driven by the upregulation of transcription factors such as SNAIL (SNAIL1), SLUG (SNAIL2), TWIST1, and FOXM1. Our study is the first to demonstrate that FSH promotes EndMT in endothelial cells via the upregulation of FOXM1 and SNAIL. Previous research has shown that FSH induces EMT in ovarian cancer cells through the FSHR-PI3K/Akt-Snail signaling pathway [36]. Additionally, FOXM1, an oncogenic transcription factor highly expressed in various cancers—including skin, colon, and breast cancers—induces EMT by modulating key molecules such as cyclin B1, Slug, and Snail, thereby promoting tumor cell proliferation and migration [37–39]. Given the similarities between EndMT and EMT, these insights underscore the scientific validity and rationale of our research. Furthermore, our study is the first to provide evidence that FSH enhances FOXM1 protein expression through posttranscriptional regulation, offering new insights into the effects of FSH on both EMT and EndMT.

The dynamic m6A modification system, governed by writers (methyltransferases), erasers (demethylases), and readers (binding proteins), critically regulates EMT [40, 41] and is emerging as a key modulator of EndMT [42, 43]. ALKBH5, an m6A eraser, facilitates EMT through TGFβ/SMAD [44], tumor protein p53 (p53) [45], and yes-associated protein (YAP) signaling [46], with documented involvement in FSH-driven ovarian cancer EMT via Snail m6A demethylation [47]. Notably, ALKBH5 has also been implicated in cardiovascular diseases. For instance, Han et al. demonstrated its vital role in cardiomyocyte proliferation and heart regeneration [48], while Kumari et al. highlighted its importance in maintaining angiogenesis in endothelial cells following acute ischemic stress [16]. However, the impact of ALKBH5 on EndMT has not been previously reported. This study is the first to reveal that ALKBH5 serves a critical function in FSH-promoted plaque instability by regulating the EndMT process, offering a new perspective for future research on plaque instability.

The study found that FSH upregulates ALKBH5, leading to a reduction in the m6A level of *FOXM1* mRNA, which enhances the binding of the HuR protein to *FOXM1* mRNA and increases the stability of *FOXM1* mRNA, thereby promoting its protein expression. In glioma cells, ALKBH5 demethylates *FOXM1* mRNA at m6A sites within the 3′-UTR regions, facilitating the binding of the RNA-binding protein HuR to *FOXM1* pre-mRNA in the nucleus, ultimately enhancing FOXM1 expression [17]. This study further confirmed that in HUVECs, ALKBH5 demethylation not only increased HuR binding to *FOXM1* pre-mRNA but also promoted HuR binding to *FOXM1* mRNA, thus extending the half-life of *FOXM1* mRNA. This mechanism of promoting *FOXM1* expression by increasing mRNA stability was also validated in uveal melanoma cells [49].

Hu antigen R (HuR), a central RNA-binding protein in posttranscriptional regulation and a key member of the ELAVL family (HuB/C/D) [50], governs thousands of transcripts implicated in cardiovascular pathophysiology—spanning cellular proliferation, inflammation, angiogenesis, and hypoxia adaptation [51, 52]. Our findings suggest that FSH may modulate gene expression through HuR to facilitate EndMT, extending the regulation of target genes by sex hormones via RNA-binding proteins. Similar regulatory patterns include, for example, estrogen (E2) elevating AUF1 p45 to stabilize *ERα* mRNA in uterine tissue [53]. In addition, the phytoestrogen calycosin disrupts HuR-*lncRNA HOTAIR* binding in breast cancer [54]. This evidence underscores the regulatory role of FSH in RNA-binding proteins and opens new avenues for understanding FSH’s mechanisms of action.

While the functions of m6A enzymes are well studied, their upstream regulatory mechanisms remain less understood. Research has shown that ALKBH5 activity is modulated by K235 acetylation and its interaction with the regulatory subunit PSPC1, which enhances its demethylase activity and oncogenic potential [55]. In head and neck squamous cell carcinoma (HNSCC) cells, RNA-binding motif protein 33 (RBM33) recruits ALKBH5 to m6A-marked substrates and activates its demethylase activity by removing SUMOylation [56]. In leukemia stem cells (LSCs), KDM4C enhances *ALKBH5* level by elevating chromatin accessibility at the ALKBH5 locus, diminishing H3K9me3 levels, and facilitating the binding of MYB and Pol II [57]. These results illustrate the diverse regulation of ALKBH5. However, regulation at the gene promoter level is also crucial. In testicular stromal cells, human chorionic gonadotropin (HCG) enhances the accumulation of transcription factors CEBPB and TFEB at the *ALKBH5* promoter, thereby increasing ALKBH5 expression [58]. Similarly, our investigation into the molecular mechanisms of FSH revealed that FSH stimulates the accumulation of p-CREB Ser129/133 at specific binding sites within the *ALKBH5* promoter, exhibiting significant transcriptional activity and further substantiating the role of transcription factors in regulating ALKBH5 expression.

FSH is widely recognized to bind its receptor (FSHR), activating it and subsequently coupling with the Gαs subunit to initiate the cAMP/PKA signaling pathway. This activation results in the phosphorylation of CREB and its binding to target gene promoters, thereby modulating gene expression [27]. Recent studies have expanded this understanding, revealing that FSH–FSHR interactions can also activate several non-Gαs-dependent signaling pathways. For example, FSHR can couple with Gαi, Gαq, β-arrestins, or APPL1, leading to the activation of various kinase-mediated pathways, including IP3, Akt, and ERK1/2. Our findings confirm that FSH induces CREB phosphorylation via the PKA/Akt pathway, consistent with classical signaling mechanisms observed in other cell types.

Recent studies have demonstrated that EndMT is a dynamic and stage-specific pathophysiological process [59]. On the one hand, endothelial cells may transiently reside in an intermediate state of partial EndMT, and angiogenesis is a unique example of partial EndMT [60]. On the other hand, its bidirectional plasticity is evidenced by reversible phenotypic changes under transient stimuli [59], whereas persistent pathological insults (e.g., sustained inflammatory cytokine exposure or metabolic dysregulation) drive cells through intermediate states toward a robust mesenchymal transition, ultimately culminating in irreversible complete EndMT [61]. The dynamism of EndMT poses significant challenges for in vivo experimental interrogation, particularly in resolving spatiotemporal regulation of transitional states. While our current mechanistic studies focus on molecular targets in controlled in vitro systems, we will explicitly note that future investigations using lineage-tracing models and dynamic in vivo imaging will be essential to delineate the temporal regulation of FSH-mediated EndMT in pathophysiological contexts. This addition will not only strengthen the translational relevance of our findings but also clarify the in vivo contribution of FSH to EndMT-driven pathophysiology, thereby bridging mechanistic insights with therapeutic development.

Despite these limitations, the current data clearly illustrate FSH’s role in regulating the FOXM1/Snail axis through sequential signaling cascades, thereby promoting EndMT and plaque instability. These findings offer valuable insights into the increased incidence of cardiovascular events in postmenopausal women and highlight potential therapeutic targets for prevention and intervention.

## Conclusions

Our study elucidates the role and underlying molecular mechanisms by which FSH contributes to plaque instability, and it demonstrates that targeting the ALKBH5/FOXM1/SNAIL axis may be a potential therapeutic target for the prevention and intervention of cardiovascular events in postmenopausal women.

## Supplementary Information

Supplementary Material 1: Fig. S1. FSH promotes ALKBH5 mRNA expression *via* CREB signaling in HUVECs. Table S1. Serum levels of 17β-estradiol and FSH in mice groups. Table S2. Primer sequences for real-time PCR. Fig. S2-9. The uncut raw gel of WB in figures. Figure S10 The raw gel of agarose electrophoresis in figure 5/6.

## Data Availability

The complete information required to assess the findings in this investigation can be found within the manuscript and its Supplementary Materials. The authors will provide relevant datasets upon receiving formal requests.

## Abbreviations

FSH: Follicle-stimulating hormone
EndMT: Endothelial-to-mesenchymal transition
*ApoE*^−^^/^^−^: Apolipoprotein E-deficient
HUVECs: Human umbilical vascular endothelial cells
FOXM1: Forkhead box protein M1
ALKBH5: AlkB homolog 5, RNA demethylase
CVD: Cardiovascular disease
E2: Estrogen
ERT: Estrogen replacement therapy
m6A: MRNA *N*^6^-methyladenosine
