# Supplementary material for: Follicle-stimulating hormone promotes EndMT in endothelial cells by upregulating ALKBH5 expression
Source: Cell Mol Biol Lett. 2025 Apr 4;30:41. doi: 10.1186/s11658-025-00720-y (PMC11969750; doi:10.1186/s11658-025-00720-y)
Supplement: Supplementary file 1 — Supplementary Material 1: Fig. S1. FSH promotes ALKBH5 mRNA expression via CREB signaling in HUVECs. Table S1. Serum levels of 17β-estradiol and FSH in mice groups. Table S2. Primer sequences for real-time PCR. Fig. S2-9. The uncut raw gel of WB in figures. Figure S10 The raw gel of agarose electrophoresis in figure 5/6. [file 11658_2025_720_MOESM1_ESM.docx]

# Supplementary Information

# Follicle Stimulating Hormone Promotes EndMT in Endothelial Cells by Up-regulating ALKBH5 Expression

**Ping Li^1, 2†^, Yixiao Xiang ^2†^，Jinzhi Wei^2†^, Xingyan Xu^2^, Jiale Wang^2^, Haowei Yu^2^, Xiaosa Li ^2, 3*^, Huiping Lin ^2, 3*^,** **Xiaodong Fu^1, 2, 3*^**

1. Department of Cardiovascular, Affiliated Qingyuan Hospital, The Sixth Clinical Medical School, Guangzhou Medical University, Qingyuan People’s Hospital, Qingyuan, Guangdong, 511518, P.R. China
2. Key Laboratory of Cardiovascular Diseases, Guangzhou Municipal and Guangdong Provincial Key Laboratory of Protein Modification and Degradation, School of Basic Medical Sciences, Guangzhou Medical University, Guangzhou, Guangdong, 511436, P.R. China.
3. Guangzhou Institute of Cardiovascular Disease, Guangdong Key Laboratory of Vascular Diseases, State Key Laboratory of Respiratory Disease, The Second Affiliated Hospital of Guangzhou Medical University, Guangzhou, Guangdong, 510260, P.R. China.

***Corresponding author Email:** Xiaodong Fu, Email: fuxiaod@gzhmu.edu.cn; Huiping Lin, Email: 2021390022@gzhmu.edu.cn; Xiaosa Li, Email: xiaosali@gzhmu.edu.cn.

† These authors contributed equally to this work.

# This word file includes:

• Supplementary Figures and Figure legends

• Supplementary Tables

# Figure S1

**Figure S1 FSH promotes ALKBH5 mRNA expression *via* CREB signaling in HUVECs.** HUVECs were transfected with two different shRNAs and treated with FSH, than the protein levels of FOXM1 and ALKBH5 were examined by Western blotting (A and B). HUVECs were treated with PI3K inhibitor Wortmannin (WM, 1μM), MEK inhibitor PD98059 (PD, 20μM), PKA inhibitor H89 (10μM) and CREB inhibitor 666-15 (10μM) respectively, and then treated with FSH (100 mIU/mL) for 24 h. ALKBH5 mRNA level was detected by qRT-PCR (E). Statistical analysis was conducted using two-way ANOVA with Dunnett's multiple comparisons test. n ≥ 3, * p < 0.05 vs. CON, # p < 0.05 vs. FSH.

# Table S1

**Serum levels of 17β-estradiol and FSH in mice groups.**

|  | SHAM  (n=10) | OVX  (n=10) | OVX+E2  (n=9) | OVX+E2+FSH  (n=7) |
| --- | --- | --- | --- | --- |
| E2 (pg/mL) | 45.18±10.76 | 9.78±3.10** | 52.54±8.71 | 47.02±5.50 |
| FSH (ng/mL) | 7.17±2.88 | 12.80±4.25** | 8.10±3.17 | 17.37±3.43## |
| All data are presented as mean ± SD. Statistical analysis was performed using one-way ANOVA with Sidak's multiple comparisons test, with n = 7 - 10, ** p < 0.01 vs. SHAM group; ## p < 0.01 vs. OVX + E2 group | | | | |

# Table S2 Primer sequences for real-time PCR

| **Gene** | **Primer (5’ to 3’)** |
| --- | --- |
| SNAIL-F | GGCAATTTAACAATGTCTGAAAAGG |
| SNAIL-R | GAATAGTTCTGGGAGACACATCG |
| FOXM1-F | TGCAGCTAGGGATGTGAATCTTC |
| FOXM1-R | GGAGCCCAGTCCATCAGAACT |
| SLUG-F | CGAACTGGACACACATACAGTG |
| SLUG-R | CTGAGGATCTCTGGTTGTGGT |
| TWIST1-F | CTCAAGAGGTCGTGCCAATC |
| TWIST1-R | CCCAGTATTTTTATTTCTAAAGGTGTT |
| ALKBH5-F | ATCCTCAGGAAGACAAGATTAG |
| ALKBH5-R | TTCTCTTCCTTGTCCATCTC |
| pre-FOXM1-F | CATAGCAAGCGAGTCCGCAT |
| pre-FOXM1-R | TGCAAGCTGAAGGTCCAACA |
| GAPDH-F | GTCAGTGGTGGACCTGACCT |
| GAPDH-R | TGCTGTAGCCAAATTCGTTG |
| ALKBH5-S1-F | CGACCCCCATTTGTTGGAG |
| ALKBH5-S1-R | GCTGCAGAGAGCCGAGCT |
| ALKBH5-S2-F | TTCTTCCAGAGTCCCCGTAGG |
| ALKBH5-S2-R | TTGGCGTCGAGCCTCCTT |
| ALKBH5-S3-F | CCGTCACCGGGACGTGA |
| ALKBH5-S3-R | TGTCGGGGTACAAGCCAGC |
| ALKBH5-S4-F | CAAAGGGGGAGGTAACGGA |
| ALKBH5-S4-R | CGCTCATATTGCCAACAGTGTC |

# Figure S2 The uncut raw gel of WB in figure 2D.

# Figure S3 The uncut raw gel of WB in figure 3A/C/E.

# Figure S4 The uncut raw gel of WB in figure 3F/H/I.

# Figure S5 The uncut raw gel of WB in figure 4D/E.

# Figure S6 The uncut raw gel of WB in figure 4G.

# Figure S7 The uncut raw gel of WB in figure 4K/L.

# Figure S8 The uncut raw gel of WB in figure 6C/D/E.

# Figure S9 The uncut raw gel of WB in figure 6F/G and figure S1A/B.

# Figure S10 The raw gel of agarose electrophoresis in figure 5/6.
